# Myelodysplastic syndromes current treatment algorithm 2018

**DOI:** 10.1038/s41408-018-0085-4

**Published:** 2018-05-24

**Authors:** David P. Steensma

**Affiliations:** Dana-Farber Cancer Institute and Harvard Medical School, Boston, Massachusetts USA

## Abstract

Myelodysplastic syndromes (MDS) include a group of clonal myeloid neoplasms characterized by cytopenias due to ineffective hematopoiesis, abnormal blood and marrow cell morphology, and a risk of clonal evolution and progression to acute myeloid leukemia (AML). Because outcomes for patients with MDS are heterogeneous, individual risk stratification using tools such as the revised International Prognostic Scoring System (IPSS-R) is important in managing patients—including selecting candidates for allogeneic hematopoietic stem cell transplantation (ASCT), the only potentially curative therapy for MDS. The IPSS-R can be supplemented by molecular genetic testing, since certain gene mutations such as *TP53* influence risk independent of established clinicopathological variables. For lower risk patients with symptomatic anemia, treatment with erythropoiesis-stimulating agents (ESAs) or lenalidomide (especially for those with deletion of chromosome 5q) can ameliorate symptoms. Some lower risk patients may be candidates for immunosuppressive therapy, thrombopoiesis-stimulating agents, or a DNA hypomethylating agent (HMA; azacitidine or decitabine). Among higher risk patients, transplant candidates should undergo ASCT as soon as possible, with HMAs useful as a bridge to transplant. Non-transplant candidates should initiate HMA therapy and continue if tolerated until disease progression. Supportive care with transfusions and antimicrobial drugs as needed remains important in all groups.

## Overview

Myelodysplastic syndromes (MDS) are defined by ineffective hematopoiesis resulting in blood cytopenias, and clonal instability with a risk of clonal evolution to acute myeloid leukemia (AML)^1,2^. Patients with MDS collectively have a high symptom burden and are also at risk of death from complications of cytopenias and AML^3^. The goals of therapy for patients with MDS are to reduce disease-associated symptoms and the risk of disease progression and death, thereby improving both quality and quantity of life^4–6^.

Because the median age at diagnosis of MDS is ~70 years, patients frequently have comorbid conditions that may influence outcomes and treatment approaches^7,8^. For instance, patients with established cardiovascular or pulmonary disease tolerate anemia poorly, while those with germline *HFE* mutations associated with hereditary hemochromatosis may have an elevated risk of organ toxicity from iron overload with repeated red cell transfusions. Functional defects in neutrophils or platelets may result in an infection or bleeding risk that is disproportionate to the degree of cytopenias^9^.

Three drugs have been approved by the Food and Drug Administration (FDA) for use in MDS-related indications: the orally administered immunomodulatory drug lenalidomide, and two parenterally administered nucleoside analogs that are DNA hypomethylating agents (HMA), azacitidine, and decitabine. In addition to these agents, there is extensive off-label use of the erythropoiesis-stimulating agents (ESA) epoetin and darbepoetin in MDS, which is supported by National Comprehensive Cancer Network (NCCN) guidelines and therefore reimbursed by Medicare as a compendium use^10,11^. No new drugs have been approved for MDS-related indications since decitabine’s FDA approval in 2006^12^, underscoring the importance of clinical trial enrollment; currently only a small proportion of patients with MDS are enrolled in prospective interventional studies.

## Risk stratification

The most commonly used tool for risk stratification in MDS is the 2012 revised International Prognostic Scoring System (IPSS-R; Table 1), which was based on a multivariate assessment of clinicopathological variables and outcomes in more than 7000 patients, conducted by the International Working Group for Prognosis in MDS (IWG-PM)^13^. The IPSS-R takes into account the number and degree of cytopenias, proportion of blasts in the marrow, and risk of the specific cytogenetic abnormalities present. The IPSS-R stratifies patients into 5 risk groups; collectively, the 2 lowest risk groups are often referred to as ‘lower risk MDS’, while the 2 higher risk groups are described as ‘higher risk MDS’.

**Table 1:** 2012 revised international prognostic scoring system for MDS (IPSS-R)

Parameter	Categories and Associated Scores (Scores in italics)				
Cytogenetic risk group^a^	Very good	Good	Intermediate	Poor	Very Poor
	*0*	*1*	*2*	*3*	*4*
Marrow blast proportion	≤2.0%	>2.0–<5.0%	5.0–<10.0%	≥10.0%	
	*0*	*1*	*2*	*3*	
Hemoglobin	≥10 g/dL	8–<10 g/dL	<8 g/dL		
	*0*	*1*	*1.5*		
Absolute neutrophil count	≥0.8 × 10^**9**^/L	<0.8 × 10^**9**^/L			
	*0*	*0.5*			
Platelet count	≥100 × 10^9^/L	50–100 × 10^9^/L	<50 × 10^9^/L		
	*0*	*0.5*	*1*		

Risk group	Total score^b^	Proportion of patients in category (%)	Median survival (survival data based on *n* = 7012) (years)	Time until AML progression (AML data available based on *n* = 6485) (years)
Very low	0–1.0	19	8.8	Not reached
Low	1.5–3.0	38	5.3	10.8
Intermediate	3.5–4.5	20	3.0	3.2
High	5.0–6.0	13	1.5	1.4
Very high	>6.0	10	0.8	0.7

^a^ Cytogenetic risk group, very good: -Y, del(11q); good: normal; del(5q) ± 1 other abnormality del(20q), or del(12p); intermediate: + 8, i(17q), del(7q), + 19, any other abnormality not listed including the preceding with 1 other abnormality; poor: −7 ± del(7q), inv(3)/t(3q)/del(3q), any 3 separate abnormalities; very poor: more than 3 abnormalities, especially if 17p is deleted or rearranged

^b^Sum scores on a 0–10 point scale

Source: adapted from Greenberg P et al, *Blood* 120(12):2454–65

The intermediate risk IPSS-R group is heterogeneous, with some patients having a more indolent natural history similar to lower risk MDS and others more aggressive disease. This heterogeneity can be partly resolved by the use of molecular genetic sequencing. While a number of series have been published assessing the prognostic importance of individual mutations, the IWG-PM is currently assessing the utility of specific mutations in more than 3000 patients^14,15^. Among DNA sequencing series already reported, *TP53, NRAS, ASXL1*, and *EZH2* mutations are consistently associated with poor outcomes while *SF3B1* mutations are associated with more favorable outcomes^16–18^.

The IPSS-R has several limitations in addition to its lack of molecular genetic information. For example, the IPSS-R is only validated for adult patients with de novo disease at the time of diagnosis, and it describes expected outcomes for those treated with supportive care alone^19,20^. Future prognostic tools will hopefully be validated for a broader range of patients and will be dynamic, able to be applied at any time in the disease course.

Patients with therapy-related disease (t-MDS) associated with prior genotoxic exposure frequently have *TP53* mutations or *PPM1D* mutations and a complex karyotype^21^. These patients were excluded from the IPSS-R and should be considered to have very high risk disease.

Some patients with MDS have features of myeloproliferative neoplasms, such as monocytosis (especially in chronic myelomonocytic leukemia) or extensive marrow fibrosis. These patients have a distinct pathbiology, with over-representation of mutations such as *JAK2, SRSF2, SETBP1, CSF3R*, and *BCOR* compared to patients with MDS without proliferative features, and require a specialized treatment approach that is not discussed further here^22,23^. Patients with secondary MDS after another marrow failure syndrome (acquired aplastic anemia, paroxysmal nocturnal hemoglobinuria, or a germline disorder like Fanconi anemia or dyskeratosis congenita) also require a specially tailored approach.

## Lower risk MDS

Some patients with MDS have mild cytopenias and are asymptomatic at the time of diagnosis. Early treatment of MDS is not known to be beneficial in terms of preventing clonal evolution or death. Therefore, observation is appropriate for asymptomatic lower risk patients until their cytopenias worsen or they become more symptomatic.

**Fig. 1:**
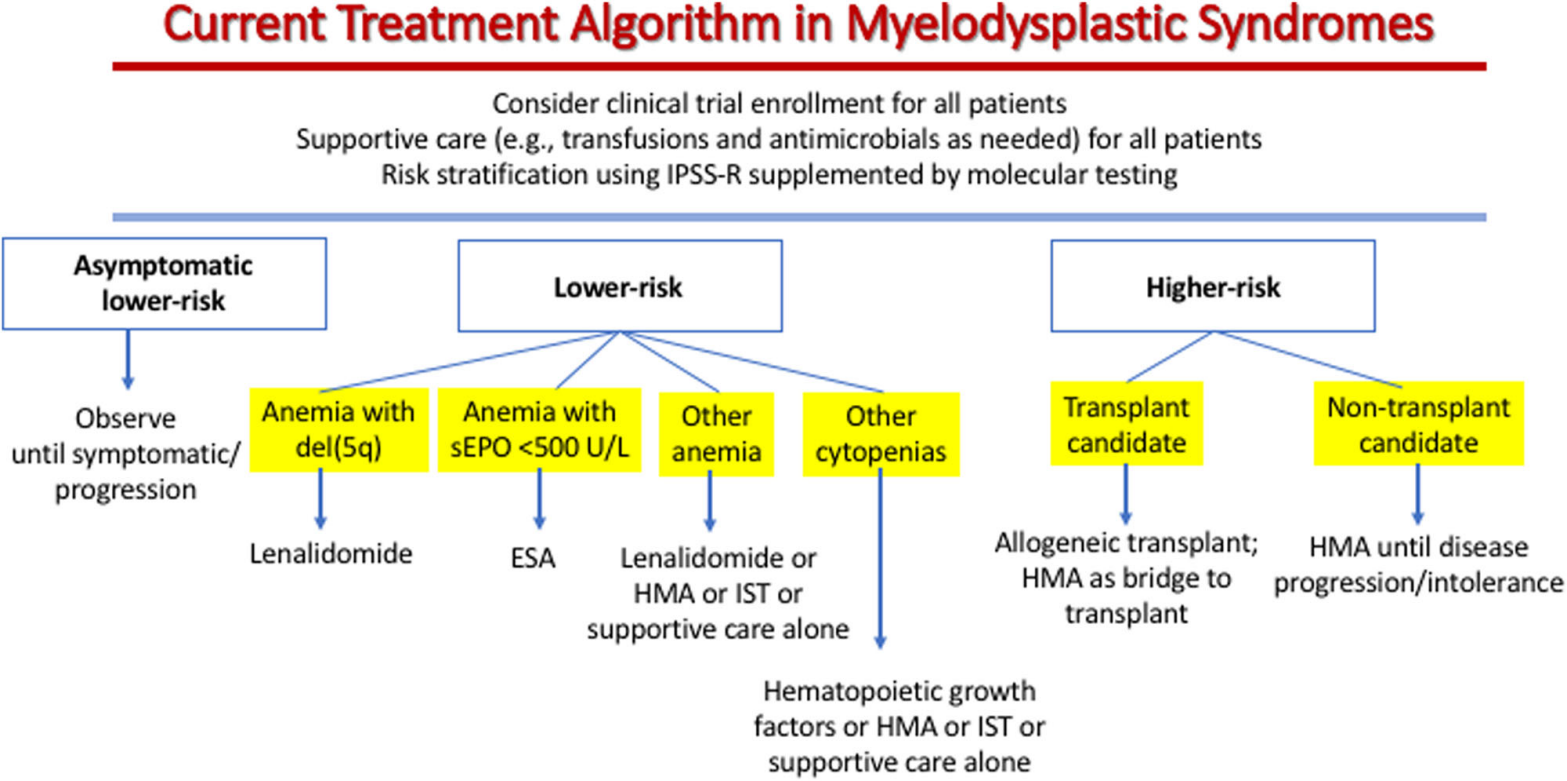
MDS treatment algorithm as described in the text. Clinical trials should be considered for all patients, but is recognized that many patients will not have access to trials or will not be eligible for available trials or will not want to go on trials, especially those requiring travel to a major center. In fact only a very small proportion of patients with MDS are currently enrolled on prospective interventional trials. However, increased trial enrollment is an important goal given the continued poor outcomes with MDS. EPO erythropoietin, ESA erythropoiesis-stimulating agent, HMA DNA hypomethylating agent, IST immunosuppressive therapy (anti-thymocyte globulin, cyclosporine, or tacrolimus)

For patients with lower risk disease and anemia associated with MDS, two parameters are important in treatment choice (Fig. 1). First, the serum erythropoietin (sEPO) level reflects endogenous renal response to anemia and is a strong predictor of the likelihood of clinical response to ESA^24^. Patients with lower risk MDS who have a sEPO < 100 U/L have a greater than 70% chance of responding to ESA, while for those patients with sEPO > 500 U/L, a trial of ESA is usually not warranted because the response rate is <10%. Second, the presence of a clonally restricted deletion of the long arm of chromosome 5 including band q31 (del5q) is associated with a high erythroid response rate to lenalidomide (65–70% transfusion independence, and 30–40% cytogenetic remission)^25,26^. Patients with a complex karyotype that includes del5q and those with excess marrow blasts respond less well to lenalidomide treatment than those whose disease lacks these features. Lower risk MDS patients with anemia who lack del5q have a 26% response rate to lenalidomide therapy, and a brief trial of lenalidomide is reasonable in such patients, although this usage is off-label^27,28^.

For patients with lower risk MDS who have other severe cytopenias beyond anemia, the most appropriate treatment approach is less clear^29^. Neutropenia in patients with MDS often does respond to use of myeloid growth factors, but these have never been shown to improve survival in MDS and have minimal effect on reducing infection risk^11^. The thrombopoiesis-stimulating agents (thrombopoietin (TPO) mimetics) eltrombopag (oral) or romiplostim (injectable) can reduce platelet transfusion needs and clinically significant bleeding events in some patients with severe thrombocytopenia^30–33^. Although a randomized trial of romiplostim in lower risk MDS was discontinued early by a data safety monitoring committee because of excess leukemia and other disease progression in the active treatment arm, a more recent update with five-year follow-up shows no difference in outcomes between patients treated on that study with romiplostim or placebo^34^. A randomized trial with eltrombopag in combination with azacitidine in a similar patient population also showed a slight increase in progression in the active treatment arm^35^. The dose of TPO mimetic required to improve platelet count is typically higher in MDS than in the labeled indication of immune thrombocytopenia^36^.

Because growth factors have somewhat limited efficacy in MDS, anti-T cell immunosuppressive therapy (IST; e.g., antithrombocyte globulin, corticosteroids, and cyclosporine or tacrolimus) or HMA are frequently considered for these patients, especially after failure of ESA or lenalidomide. Selection of appropriate patients for IST is challenging, as published reports are inconsistent about parameters that predict a higher likelihood of response; however, excess blasts, therapy-related disease, and a complex or monosomal karyotype predict lower likelihood of response to IST^37^.

A recent multicenter analysis of more than 300 MDS patients treated with IST other than corticosteroid monotherapy demonstrated that marrow cellularity less than 20% and blast proportion less than 5% were associated with a higher likelihood of transfusion independence and improved survival after immunosuppressive therapy^38^. In this multicenter series, information about mutations that might predict response to IST such as BCOR was not consistently available, but the presence of a paroxysmal nocturnal hemoglobinuria (PNH) clone or HLA DR15 status, which had been found to be of value in predicting response in smaller (mostly single-center) series, did not predict IST response. A trial of IST is reasonable in a lower risk patient who lacks excess blasts or a complex karyotype and who either has (1) anemia that has not responded to ESA or lenalidomide, or has (2) another severe cytopenia that either has not responded to growth factors or where the clinician elects not to use growth factors or the patient’s insurance will not pay for growth factors. IST is a particularly attractive consideration if the marrow is hypocellular for age.

Recently, results were reported from a multicenter adaptive randomization clinical trial of a reduced schedule HMA (i.e., three days of azacitidine 75 mg/m2/d per month, or three days of decitabine 20 mg/m2/d per month) in lower risk MDS and MDS/MPN^39^. The overall response rate in this series was higher with decitabine (32% transfusion independence rate and 70% overall response rate) than with azacitidine (16% transfusion independence rate and 49% overall response rate), so the adaptive randomization resulted in 65% of enrolled patients being treated with decitabine compared to 35% with azacitidine. It is possible that three days of azacitidine represents underdosing even in lower risk patients and a randomized trial (NCT02269280) comparing five days of azacitidine to three days of decitabine or a supportive care strategy is ongoing. The optimal dose and schedule of HMA in both higher risk and lower risk MDS is unknown, and the doses and schedules of decitabine and azacitidine used for patients with higher risk MDS are myelosuppressive and may have a less favorable risk-benefit balance for those patients with lower risk MDS. Of note, this trial of ‘lower risk’ patients enrolled 18% patients with t-MDS and 19% with MDS/MPN overlap syndromes, who should not be considered lower risk. Clinical experience has shown that patients who fail to respond to HMA rarely respond to IST, so IST after HMA is unlikely to be of value.

Luspatercept is a fusion protein that binds to ligands of the activin II receptor, altering transforming growth factor beta signaling and resulting in improved erythropoiesis in some patients with lower risk MDS^40^. A randomized trial of luspatercept versus placebo in patients with MDS and ring sideroblasts (MEDALIST; NCT02631070) completed accrual in 2017; if this trial is positive, it is likely that luspatercept will be the next drug FDA approved for MDS, and would be useful in lower risk patients with anemia and ring sideroblasts. Luspatercept has not yet been well studied in patients without ring sideroblasts.

## Higher risk MDS

For patients with higher risk MDS, the first question that must be answered is whether the patient is a candidate for allogeneic hematopoietic stem cell transplantation (ASCT). Two mathematical modeling analyses based on Center for International Blood and Marrow Transplant Research (CIBMTR) data—one analysis focused on conventional myeloablative transplant, the other inclusive of patients aged 60–70 years treated with a reduced-intensity conditioning (RIC) approaches—show that life expectancy is improved by early transplant in this subgroup, if feasible^41,42^. Increased availability of alternate donors, including haploidentical donors and cord blood, mean that larger proportion of patients have the opportunity to undergo SCT. Patients up to age 75 routinely undergo transplant now, and in the future age may not be a limitation to ASCT if performance status remains excellent.

While details about conditioning regimens for ASCT, graft-versus-host prophylaxis and management, and post-transplant infectious disease prophylaxis and pre-emptive management are beyond the scope of this review, a recent CIBMTR/Clinical Trials Network randomized trial of RIC compared with myeloablative conditioning (MAC) is worth noting^43^. This study enrolled patients aged 18–65 years with a low transplant comorbidity index and <5% marrow blasts, and randomly assigned them to receive MAC (*n* = 135) or RIC (*n* = 137) followed by ASCT from HLA-matched related or unrelated donors. While overall survival at 18 months was slightly higher with MAC, this difference was not statistically significant. RIC resulted in lower treatment-related mortality but at the cost of higher relapse rates compared with MAC. These data support the use of MAC as the standard of care for patients with MDS who are able to tolerate more intense conditioning.

It is not clear whether treatment of higher risk MDS prior to ASCT is beneficial, but pre-transplant bridging therapy is often considered to cytoreduce disease, especially for those patients who are going to go on to get RIC approaches and those who have more than 10% marrow blasts^44^. Pre-transplant cytoreductive therapy can also be considered in those for whom there will be a delay in transplant due to lack of donor availability or insurance approval. In the past, intensive AML-type induction chemotherapy was commonly used for pre-transplant cytoreduction in MDS, but today HMA therapy is used far more frequently and outcomes are at least as good with HMA as with intensive chemotherapy^45^.

For those patients who are not transplant candidates, HMA therapy is most appropriate. In a randomized trial of 358 higher risk MDS patients, azacitidine treatment was associated with a median survival of 24 months compared to 15 months in patients treated with intensive chemotherapy, low-dose cytarabine, or best supportive care^46^. A similar survival improvement has not been seen in decitabine randomized studies, but these trials enrolled a higher risk subgroup of patients and treated patients for a shorter period of time, so that the trials are not comparable to the azacitidine study. These agents are considered comparable by most clinicians, with treatment choice left up to individual providers based on considerations such as regional licensing of drug or local cost considerations. Some studies of ‘real world’ experience with azacitidine have not been able to replicate the survival outcomes seen in the randomized trial, so the actual benefits of HMA may be less than has been commonly presented^47^.

The optimal dose and schedule for each HMA is unclear; the best studied schedule of azacitidine is 75 mg/m^2^ per day for seven days every 28 days, whereas the most commonly used decitabine schedule for higher risk MDS is 20 mg/m^2^ per day for five days every 28 days, which avoids weekend dosing^48–50^. Lower doses have been studied in MDS but are not approved from a regulatory standpoint, and it is unclear if response rates mirror that seen with the most widely studied regimens. Recently, a very high response rate was reported with 10-day decitabine treatment in patients with higher risk MDS or AML associated with a *TP53* mutation^51^. It is not clear, however, if 5-day decitabine or azacitidine would be as effective as 10 day decitabine, and 10 day decitabine is highly myelosuppressive with a high frequency of opportunistic infections.

HMA can decrease clonal burden and may therefore result in improved hematopoiesis, but do not eradicate transformed stem cells, so relapse is inevitable. The mechanism of action of HMA is unclear, and may result from a combination of conventional cytotoxic, DNA hypomethylation (no specific signature), and immune-related mechanisms including changes in interferon signaling and presentation of neoantigens as epitopes to the immune system^52,53^. This has led to difficulty in predicting responders, and available molecular genetic assays do not differentiate responders versus non-responders to an extent that influences treatment selection.

Once an HMA fails the patient, either via intolerance, resistance, or relapse after favorable response, there is no approved second-line therapy and the outlook is poor with a median survival of less than six months^54–56^. Addition of the bcl-2 inhibitor venetoclax (an off-label use) can recapture response and a subset of patients for whom HMA alone is inadequate, and ongoing trials are evaluating venetoclax both after HMA failure (NCT02966782) and in treatment-naïve patients (NCT02942290)^57^. The immune checkpoint (PD1, PDL1, CTLA4) inhibitors, despite their high rate of efficacy in certain solid tumors, are of limited use as monotherapy in MDS, but are being evaluated in more than 10 different trials as combination therapy with HMA^58^. Other combination approaches including lenalidomide plus azacitidine and histone deacetylase inhibitors plus HMA have not resulted in improved outcomes compared to HMA alone despite in vitro synergy, largely due to excess myelosuppression and early treatment withdrawal^59–62^. In January 2018, it was announced that the ongoing INSPIRE trial of the multikinase inhibitor rigosertib versus best supportive care in IPSS-R high and very high risk patients after HMA failure (NCT02562443) would increase its accrual goal because of promising early results. If the results are favorable, rigosertib would be the first drug approved for second-line therapy in MDS.

## Supportive care

Support of patients with severe symptomatic anemia with red cell transfusions and severe thrombocytopenia with platelet transfusions is a mainstay of therapy for MDS. Fevers in patients with MDS must be taken seriously, and antimicrobial protocols for febrile neutropenia followed carefully, as infection is the leading cause of death in MDS^63,64^. The benefit of prophylactic antimicrobials is controversial.

For patients with thrombocytopenia who are refractory to platelet transfusions, the TPO agonists mentioned above may be of help, and the antifibrinolytic agents epsilon-aminocaproic acid or tranexamic acid can reduce bleeding in some of those with recurrent mucosal hemorrhage^65^. Androgens such as danazol or oxymetholone may improve hemoglobin or platelet count in a minority of patients, but liver tests must be monitored during therapy and some older men may have difficulty with urinary retention due to an increase in prostate hyperplasia^66^.

Patients who receive repeated transfusions with red cells may accumulate excess iron, which can cause tissue injury via generation of reactive free radicals. Iron chelation therapy with deferasirox or deferoxamine may be helpful in selected cases, but the effect of chelation on outcomes is controversial and such therapy is expensive (deferasirox) or inconvenient (deferoxamine)^67^. A randomized trial of deferasirox versus placebo in lower risk MDS patients with red cell transfusion dependence and a serum ferritin greater than 1000 ng/mL (TELESTO; NCT00940602) has completed accrual and results are eagerly awaited.

### Future directions

Dozens of novel agents are in development for MDS, as recently reviewed by Brunner and Steensma^68^. These include luspatercept, rigosertib, immune checkpoint inhibitors, and venetoclax, as mentioned above. In addition, three new HMAs are in late phase clinical trials: guadecitabine (a parenterally administered dinucleotide nucleoside analog formerly known as SGI-110), CC486 (an orally bioavailable form of azacitidine), and cedazurine (formerly known as ASTX727, an orally administered fixed-dose combination of decitabine and a cytidine deaminase inhibitor). The oral HMAs may allow exploration of extended doses and schedules compared to the available parenteral agents. Some patients who were failed by available HMAs responded to guadecitabine; a phase 3 study is ongoing in relapsed/refractory disease (NCT02907359)^69^. Imetelstat, a telomerase inhibitor also being studied in myeloproliferative neoplasms, is in a Phase 2 trial (NCT02598661) and transfusion independence rates were ~30% in the first phase of this study^70^. A major priority is restoration of TP53 activity given the poor prognosis of patients with mutant or deleted TP53, and trials (NCT03072043) are ongoing with APR-246, a TP53 modulator, and other agents in this subgroup.

## Conclusion

Treatment of patients with MDS requires appropriate risk stratification and a tailored approach that depends on the specific pattern and degree of cytopenias, sEPO level, presence of del(5q), marrow cellularity, and age and comorbid conditions. New approaches are needed, as the majority of patients diagnosed with MDS in 2018 will die of complications of cytopenias^64^.
